# Burden of neurodevelopmental disorders in low and middle-income countries: A systematic review and meta-analysis

**DOI:** 10.12688/wellcomeopenres.13540.3

**Published:** 2018-03-14

**Authors:** Mary Bitta, Symon M. Kariuki, Amina Abubakar, Charles R.J.C Newton

**Affiliations:** 1 KEMRI-Wellcome Trust Research Programme, Centre for Geographic Medicine Research , (Coast), Kilifi, Kenya; 2 Department of Public Health, Pwani University, Kilifi, Kenya; 3 Department of Psychiatry, University of Oxford, Oxford, UK

**Keywords:** neurodevelopment, low and middle-income countries

## Abstract

**Background:** Childhood mortality from infectious diseases has declined steadily in many low and middle-income (LAMIC) countries, with increased recognition of non-communicable diseases such as neurodevelopmental disorders (NDD). There is lack of data on the burden of NDD in LAMIC. Current global burden of these disorders are largely extrapolated from high-income countries. The main objective of the study was therefore to estimate the burden of NDD in LAMIC using meta-analytic techniques.

**Methods:** We systematically searched online databases including Medline/PubMed, PsychoInfo, and Embase for studies that reported prevalence or incidence of NDD. Pooled prevalence, heterogeneity and risk factors for prevalence were determined using meta-analytic techniques.

**Results:** We identified 4,802 records, but only 51 studies met the eligibility criteria. Most studies were from Asia-Pacific (52.2%) and most were on neurological disorders (63.1%). The median pooled prevalence per 1,000 for any NDD was 7.6 (95%CI 7.5-7.7), being 11.3 (11.7-12.0) for neurological disorders and 3.2 (95%CI 3.1-3.3) for mental conditions such as attention-deficit hyperactivity disorder (ADHD). The type of NDD was significantly associated with the greatest prevalence ratio in the multivariable model (PR=2.6(95%CI 0.6-11.6) (P>0.05). Incidence was only reported for epilepsy (mean of 447.7 (95%CI 415.3-481.9) per 100,000). Perinatal complications were the commonest risk factor for NDD.

**Conclusion:** The burden of NDD in LAMIC is considerable. Epidemiological surveys on NDD should screen all types of NDD to provide reliable estimates.

## Introduction

Neurodevelopmental disorders (NDD) are a group of disorders that typically manifest early in development and are characterised by developmental deficits that produce impairments of personal, social, academic, or occupational functioning ^1^. They include autism spectrum disorders (ASD), attention-deficit hyperactivity disorder (ADHD), epilepsy, intellectual disability, hearing impairments, visual impairments and motor impairments including cerebral palsy, among others. Some disorders overlap, for example in children with epilepsy, ASD occurs in 22% ^2^, ADHD in 33% ^2^, and behavioural/emotional problems in 30–50% ^3, 4^. Although more than 80% of the world’s births occur in low and middle-income countries (LAMIC) ^5^, most of the epidemiology of NDD is based on data from developed countries ^6– 8^. The lack of precise epidemiological data on NDD in poorer countries affects planning of public health interventions.

In the past decade, infant mortality has declined in many LAMIC and preventing childhood morbidity is becoming a public health priority. However, there are few studies on the epidemiology of NDD in LAMIC, where the burden could be greatest because: (i) the incidence of risk factors for NDD such as perinatal complications ^9^, head injury, parasitic infections ^10^ and nutritional deficiencies are higher in LAMIC according to the global burden of disease study ^11^; (ii) following the successful control of infectious diseases, children with neurological disability are surviving ^12^.

So far, no precise estimate exists for NDD in LAMIC. Available studies focus mostly on a few conditions ^13^, are conducted in a small number of countries. In particular the Ten Questions Questionnaire (TQQ) has been used to determine the prevalence of neurological impairment and disability, but this screening tool is poor at detecting NDD such as ASD and ADHD. It is unclear if the variation in estimates is due to methodological differences or is dependent upon NDD type/condition, calling for the need to review the available studies to measure the causes of variation in estimates.

To fill the knowledge gap that exists regarding the epidemiology of NDD in LAMIC, we conducted a systematic review of studies reporting prevalence and incidence of NDD. We pooled the estimates for different types of NDD and determined the causes of heterogeneity. We also described the risk factors associated with NDD among the studies included in the burden estimates.

## Methods

### Search strategy

We searched all articles of population studies on prevalence or incidence of NDD in the electronic databases MEDLINE and EMBASE, African Index Medicus and CINHL databases. Our last search was conducted on 31/06/2017.

We included references from identified articles that met the inclusion criteria. The main search terms were (“neurodev*” and “prevalence”) or (“neurodev*” and “incidence”) with limits (humans, journal article) in MEDLINE and EMBASE ( Table 1). We used recommendations of National Health Service Centre for Reviews and Disseminations to develop a search strategy where the review question was broken down to search terms.

**Table 1.** Search terms.

((epidemiology) OR (prevalence) OR (incidence) OR (burden)) AND ((neurodevelopmental disorder*) OR (behav* problem*) OR (behav* disorder*) OR (cogniti* impairment*) OR (language difficult*) OR (learning disabilit*) OR (Hearing difficult*) OR (hearing impairment*) OR (visual impairment*) OR (psychotic disorder*) OR (hyperkinetic*) OR (psychiatric disorder*) OR (ataxia) OR (motor impairment*) OR (psychomotor disorder*) OR (attention deficit and hyperactivity disorder*) OR (autis*) OR (epilepsy) OR (cerebral palsy)) AND ((Children) OR (infant) OR (kids) OR (teen*) OR (adolescent*)) AND ((risk factor*) OR (factor*) OR (predisposing factor)) AND ((low income countr*) OR (low-income countr*) OR (middle income countr*) OR (middle-income countr*) OR (developing countr*) OR (developing nation*) OR (Africa) OR (south America) OR (asia) OR (resource poor countr*) OR (third world)) AND “humans”[MeSH Terms] AND (“0001/01/01”[PDAT] : “2017/06/31”[PDAT])

Two authors (MAB and SK) reviewed the titles and abstracts of articles obtained from online searches. We reviewed full texts of eligible articles from this initial evaluation stage. Reporting of findings followed the Preferred Reporting Items for Systematic Reviews and Meta-Analysis guidelines ^14^.

### Inclusion and exclusion criteria

All population-based studies measuring the prevalence or incidence of any of the NDD listed were included. A population denominator was an inclusion criterion for research database studies. We only considered studies with a sample population of <19 years or if results were stratified by age, and a population denominator for sample <19 years was provided. We excluded studies that were not conducted in a LAMIC as defined by the current World Bank Classification of Economies ^15^. We also excluded reviews, editorials, letters, commentaries, case series and case reports, abstracts without full texts and special-group studies, e.g., prevalence of cerebral palsy in children with a history of birth trauma, or duplicate populations. In addition, we report the findings from studies that used the TQQ, since this is the longest established screening tool and most widely reported.

### Procedures

We collected all the relevant study level information required for analysis using a data extraction template designed and piloted *a priori* by the authors. MAB and SK extracted data independently. We resolved disagreements by consensus. For included studies, we recorded information on the NDD under investigation, author, year of publication, country, study design, study population, data collection and ascertainment method (medical records or questionnaires [with physical examination] in population-based studies), age, number of cases, and the prevalence/incidence estimate. The quality of all the studies that met the inclusion criteria was investigated using the Joanna Briggs Institute Prevalence Critical Appraisal Tool ^16^.

### Statistical analysis

We tabulated crude prevalence estimates expressed per 1,000 persons and the incidence expressed per 100,000 persons per year in summary tables along with their 95% confidence intervals (95%CI), stratified according to the region where the study was conducted. Where an eligible study did not report the prevalence of NDD, we derived the prevalence through dividing the total cases reported by the total sample studied, and then expressed per 1,000 population. We obtained a range using the 5th and 95th percentiles as *m ±* 1.96τ, where *τ* is the standard deviation. The computed prevalence was then utilised in the meta-analysis approach described below. We collected data on incidence as reported in a study. To estimate pooled prevalence estimates and assess for heterogeneity, we log-transformed observed prevalence and fitted random effects models to these estimates using the “metan” command in STATA v 13.1 (StataCorp., TX). The random effects model approach is robust where there is significant heterogeneity across study estimates. It uses information on prevalence and study size. It assumes that the outcomes being estimated in the different studies are not identical, but follow a lognormal distribution, allowing for among-study variation ^17^. We then back-transformed the log estimates to the original scale to obtain prevalence estimates the confidence intervals around the estimates.

We used forest plots ^18^ to visualize heterogeneity among studies. Using the Cochran chi-square (χ2) test, we examined the null hypothesis that the observed heterogeneity was due to sampling error. We anticipated heterogeneity because of methodological differences so we quantified the degree of heterogeneity across studies using the statistic *I* ^2^, from the random effect meta-analysis model. *I* ^2^ describes the percentage of the variability in estimates that is due to true differences in prevalence rather than sampling error ^19, 20^. A value >50% is considered as substantial heterogeneity.

We investigated six study level covariates for their association with prevalence estimates: the quality score of the study, continent of the study, the year, the domain studied, the method of case identification (clinical diagnosis or screening only) and the study setting (rural/urban). We examined the influence of these variables on study prevalence using both univariate and multivariable random effects meta-regression models fitted using the “metareg” command in STATA. This approach assumes two additive components of variance, one representing the variance between studies and the other the variance within studies (i.e., error variance). The proportion of heterogeneity explained by each of the covariates was estimated by comparing the between-studies component of variance in the null model (τ _0_^2^) with the estimate of *τ* ^2^ for the model including covariates ((τ _0_^2^– *τ* ^2^)/τ _0_^2^).

## Results

### Details of eligible studies

**Figure 1.**
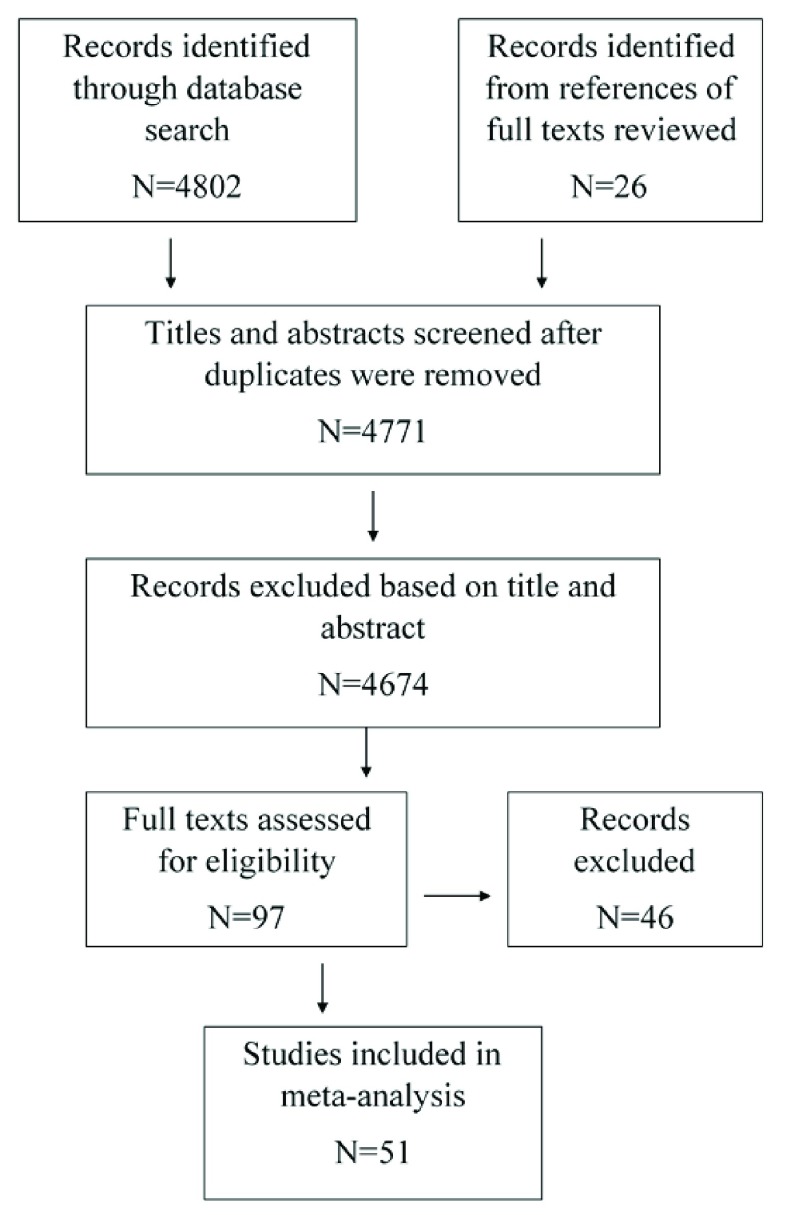
A summary of the study selection process.

Electronic database search yielded 4,802 articles of which 51 studies on a total population sample size of 2,925,139 included in the meta-analysis ( Figure 1). Majority of the studies were from Asia-Pacific region (n=27 (52.9%)) and Africa (n=16 (31.4%)), with six (11.8%) from Latin America and two (3.9%) from two or more continents. Table 2 summarized the study characteristics of included studies.

**Table 2.** Summary of study characteristics for studies included in the meta-analysis.

Author	Year of publication	Country	Study setting	Domain studied	Total sample	Overall prevalence
Wagner RG ^21^	2014	South Africa		Epilepsy	36816	2
Bevilacqua MC ^22^	2013	Brazil	Urban	Hearing impairment	218	1.4
Ngugi AK	2013	Kenya	Rural	Epilepsy	129069	3
Ngugi AK	2013	Multisite	Mixed	Epilepsy	308028	9.4
Ebrahimi H ^23^	2012	Iran	Urban	Epilepsy	568	15.8
Caca I ^24^	2013	Turkey		Visual impairment	21062	493.4
Arruda MA ^25^	2015	Brazil		Attention deficit hyperactivity disorder, emotional and behavioural problems	1830	51
Burton KJ ^26^	2012	Tanzania		Epilepsy	38523	2.9
Basu M ^27^	2011	India	Urban	Visual impairment	3002	152.2
Prasad R	2011	Brazil		Attention deficit hyperactivity disorder	4423	199
Raina SK ^28^	2011	India		Cerebral palsy	3966	2.27
Czechowicz JA ^29^	2010	Peru	Urban	Hearing impairment	355	64.8
Tasci Y ^30^	2010	Turkey		Hearing impairment	16975	2.2
Winkler AS ^31^	2009	Tanzania	Rural	Epilepsy	7399	11.2
Saldir M ^32^	2010	Turkey	Urban	Mild neurological dysfunction, cerebral palsy	169	165.7
Perera H ^33^	2009	Sri Lanka		Autism	374	10.7
Khan NZ ^34^	2009	Bangladesh	Rural	Behaviour problems	499	146
Mung'ala-odera V ^35^	2008	Kenya	Rural	Epilepsy	10218	10.7
Wong VC ^36^	2008	China	Urban	Autism spectrum disorder	1174322	1.6
Velez van meerbeke A ^37^	2007	Colombia	Urban	Neurodevelopmental delay disorders	2043	30.8
Zeidan Z ^38^	2007	Sudan	Urban	Blindness	29048	1.4
Del brutto OH ^39^	2005	Ecuador	Rural	Epilepsy	1083	5.5
Ersan EE ^40^	2004	Turkey		Attention deficit hyperactivity disorder, oppositional defiant disorder	1425	81
Serdaroglu IU ^41^ A	2004	Turkey		Epilepsy	46813	8
Wong V ^36^	2004	China		Epilepsy	1103	4.5
Mousa Thabet AA ^42^	2001	Gaza		Behavioural/emotional problems	959	481.8
Couper J ^43^	2002	South Africa	Rural	Learning disability, cerebral palsy, perceptual disability, seizure disorder	2036	17
Bulgan T ^44^	2002	Mongolia		Visual impairment	416	.2
Zainal M ^45^	2002	Malaysia		Visual impairment	8504	10.3
Onal AE ^46^	2002	Turkey		Epilepsy	903	8.9
Rao RS ^47^	2002	India	Rural	Hearing impairment	855	119
Liu XZ ^48^	2001	China		Hearing impairment	34157	6.6
Olusanya BO ^49^	2000	Nigeria	Urban	Hearing loss	359	139
Liu J ^50^	2000	China		Cerebral palsy	385185	1.5
Liu JM	1999	China	Urban	Cerebral palsy	388192	1.6
Brito GN ^51^	1999	Brazil	Urban	Attention deficit hyperactivity disorder	402	32
Hackett RJ ^52^	1997	India	Urban	Epilepsy	1172	22.2
Morioka I ^53^	1996	China	Rural	Hearing impairment	282	198.6
Okan N ^54^	1995	Turkey		Neurological disorders	5002	66
Mulatu MS ^55^	1995	Ethiopia	Urban	Psychopathology	611	270
Rwiza HT ^56^	1992	Tanzania		Epilepsy	11023	6.6
Koul R ^57^	1988	India		Epilepsy	26419	3.2
Osuntokun BO ^58^	1987	Nigeria	Urban	Epilepsy	10978	6
Bash KW	1987	South Africa	Rural	Motor impairment	1022	14.7
Taha AA ^59^	2015	Egypt	Both	Hearing loss	555	20.9
Wamithi S ^60^	2015	Kenya	Urban	Attention deficit hyperactivity disorder	240	6.3
Durkin MS ^61^	1992	Multiple	Rural	Epilepsy	22125	
Yoshito Kawakatsu ^62^	2012	Kenya	Rural	Neurological impairments *	6362	29
Shahnaz HI ^63^	2012	Pakistan	Rural	Neurological impairments	176364	5.5
Biritwum RB	2001	Ghana	Rural	Neurological impairments	2550	6.7
Singhi P ^64^	2007	India	Rural	Neurological impairments	1763	4.3
Ilyas Mirza ^65^	2008	Pakistan	Rural	Neurological impairments	1789	248

* These included epilepsy, cognition, hearing, motor and visual impairments.

### Critical appraisal of study quality

The median quality score for all the 51 eligible studies was 80% (IQR 66.7-90.0) as summarized in Table 3. Of the 51 studies, 9 (20%) fulfilled all the criteria for high quality in observational studies, with the remainder being of acceptable quality. Of these 9 studies, 6 had all the 10 criteria presents while for 3 studies, the last criteria (“Were subpopulations identified using objective criteria”) was not applicable. The range of the median age (where available) was 0.7–19.0 years. The median percentage female participants in the study was 48.5% (IQR 47.8-50.1) and they were not under-represented, compared to males (p=0.903).

**Table 3.** Critical appraisal of study articles using the Joanna Briggs Critical Appraisal Tool for Observational Studies.

Author	Year	Was the sample representative of the target population?	Were study participants recruited in an appropriate way?	Was the sample size adequate?	Were the study subjects and setting described in detail?	Is the data analysis conducted with sufficient coverage of the identified sample?	Were objective, standard criteria used for measurement of the condition?	Was the condition measured reliably?	Was there appropriate statistical analysis?	Are all important confounding factors/ subgroups/ differences identified and accounted for?	Were subpopulations identified using objective criteria?	Quality
Wagner RG	2014	Yes	Yes	Yes	Yes	Yes	Yes	Yes	Yes	Yes	Not applicable	100
Bevilacqua MC	2013	Yes	Yes	Yes	Yes	Unclear	Yes	Yes	Unclear	Unclear	Yes	70
Ngugi AK	2013	Yes	Yes	Yes	Yes	Yes	Yes	Yes	Yes	Yes	Yes	100
Ngugi AK	2013	Yes	Yes	Yes	Yes	Yes	Yes	Yes	Yes	Yes	Yes	100
Ebrahimi H	2012	Yes	Yes	Unclear	No	Unclear	Yes	Yes	Unclear	Unclear	Yes	50
Caca I	2013	Yes	Yes	Yes	No	Yes	Yes	Yes	Yes	Unclear	Yes	80
Arruda MA	2015	Yes	Yes	Yes	Yes	Yes	Yes	Yes	Yes	Yes	Yes	100
Burton KJ	2012	Yes	Yes	Yes	No	Yes	Yes	Yes	Yes	Yes	Not applicable	88.9
Basu M	2011	Yes	Yes	Yes	No	Yes	Yes	Yes	Unclear	Unclear	Yes	70
Raina SK	2011	Yes	Yes	Yes	Yes	Yes	Yes	Yes	Yes	Yes	Yes	100
Czechowicz JA	2010	Yes	Yes	Unclear	Yes	Unclear	Yes	Yes	Yes	Yes	Yes	80
Tasci Y	2010	Yes	Yes	Yes	No	Unclear	Yes	Yes	Unclear	Yes	Not applicable	66.7
Winkler AS	2009	Yes	Yes	Yes	Yes	Yes	Yes	Yes	Yes	Unclear	Not applicable	88.9
Saldir M	2010	Yes	Yes	Unclear	No	Yes	Yes	Yes	Yes	Yes	Not applicable	77.8
Perera H	2009	Yes	Yes	Yes	No	Yes	Yes	Yes	Unclear	Unclear	Not applicable	66.7
Khan NZ	2009	Yes	Yes	Yes	No	Yes	Yes	Yes	Yes	Unclear	Yes	88.9
Mung'ala- Odera V	2008	Yes	Yes	Yes	Yes	Yes	Yes	Yes	Yes	Yes	Yes	100
Wong VC	2008	Yes	Yes	Yes	Yes	Yes	Yes	Yes	Yes	Unclear	Yes	90
Velez van Meerbeke A	2007	No	No	Unclear	No	Unclear	Yes	Yes	Yes	Unclear	Yes	40
Zeidan Z	2007	Yes	Yes	Yes	Yes	Yes	Yes	Yes	Unclear	Unclear	Yes	80
Del Brutto OH	2005	Yes	Yes	Yes	Yes	Yes	Yes	Yes	Yes	Yes	Not applicable	100
Ersan EE	2004	Yes	Yes	Yes	Yes	Yes	Yes	Yes	No	Unclear	Yes	90
Serdaro?Lu A	2004	Yes	Yes	Yes	No	Yes	Yes	Yes	Yes	Unclear	Yes	80
Wong V	2004	Yes	Yes	Yes	Yes	Unclear	Yes	Yes	Unclear	Yes	Yes	80
Mousa Thabet AA	2001	Unclear	Yes	Unclear	No	Yes	Yes	Yes	Yes	Unclear	Yes	60
Couper J	2002	Yes	Yes	Yes	Yes	Unclear	Yes	Yes	Unclear	Yes	Yes	80
Bulgan T	2002	No	No	Unclear	No	Unclear	Yes	Yes	Unclear	Unclear	Yes	30
Zainal M	2002	Yes	Yes	Yes	Yes	Yes	Yes	Yes	Yes	Unclear	Yes	90
Onal AE	2002	Yes	Yes	Yes	No	Yes	Yes	Yes	Yes	No	Yes	80
Rao RS	2002	Yes	Yes	Yes	Yes	Yes	Yes	Yes	Unclear	Unclear	Not applicable	77.8
Liu XZ	2001	Yes	Yes	Yes	Yes	Yes	Yes	Yes	Yes	Unclear	Not applicable	88.9
Olusanya BO	2000	Yes	Yes	Yes	No	Yes	Yes	Yes	Yes	Unclear	Not applicable	77.8
Liu J	2000	Unclear	Unclear	Unclear	No	Yes	Unclear	Unclear	Yes	Unclear	Not applicable	22.2
Liu JM	1999	Yes	Yes	Yes	Yes	Yes	Yes	Yes	Yes	Yes	Not applicable	100
Brito GN	1999	Unclear	Yes	Unclear	No	Yes	Yes	Yes	Yes	Unclear	Yes	60
Hackett RJ	1997	Unclear	Yes	Unclear	No	Unclear	Yes	No	Unclear	No	Not applicable	22.2
Morioka I	1996	Yes	Yes	Yes	Yes	Yes	Yes	Yes	Yes	Yes	Yes	100
Okan N	1995	Yes	Yes	Yes	Yes	Unclear	Yes	Yes	Unclear	Unclear	Not applicable	66.7
Mulatu MS	1995	Yes	Yes	Yes	Yes	Yes	Yes	No	Yes	Yes	Yes	90
Rwiza HT	1992	Yes	Yes	Yes	Yes	Unclear	Yes	Yes	Unclear	No	Yes	70
Koul R	1988	Yes	Yes	Yes	Yes	Unclear	Yes	Yes	Unclear	No	Yes	70
Osuntokun BO	1987	Yes	Yes	Yes	Yes	Unclear	Yes	Yes	Unclear	Unclear	Yes	70
Bash KW	1987	yes	yes	yes	yes	unclear	yes	yes	yes	yes	Not applicable	88.9
Taha AA	2010	No	Yes	No	Yes	Yes	Yes	Yes	Yes	Yes	Yes	80
Wamithi S	2015	No	Yes	Unclear	Yes	Unclear	Yes	Yes	Yes	Unclear	Yes	60
Durkin MS	1992	Yes	Yes	Yes	Yes	Unclear	Yes	Yes	Unclear	Unclear	Not applicable	60

Each domain was marked using either “Yes”, “No”, “Unclear” or“ Not/Applicable”.

### Estimates of overall prevalence and heterogeneity

**Figure 2.**
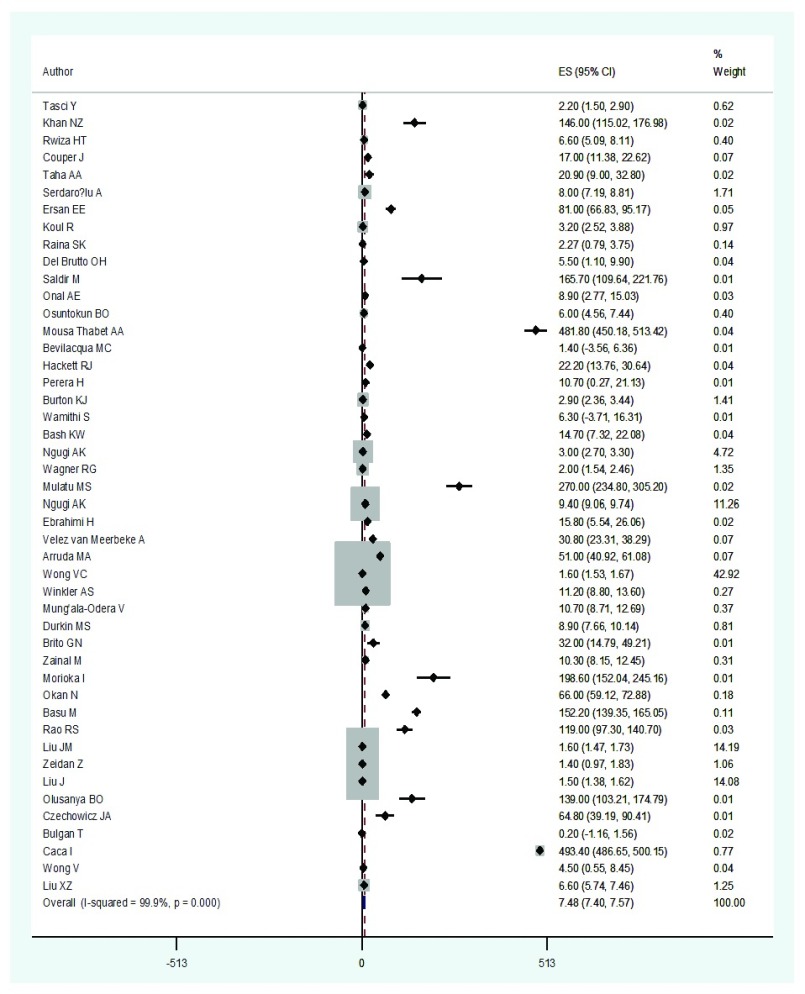
A forest plot showing the pooled median overall prevalence of all neurodevelopmental disorders in the included studies.

The pooled prevalence is reported for all the 51 studies. The pooled prevalence per 1,000 from the random effects model for any NDD was 7.5 (95% CI=7.4–7.6) ( Figure 2), 3.2 (95%CI 3.1-3.3) for mental disorders and 11.3 (95% CI 11.2-11.5) for neurological disorders. We repeated the pooled prevalence for high quality studies (quality score >80) and found a prevalence of 7.6 (95%CI 7.5-7.6) per 1,000 and for studies where cases were clinically confirmed vs those where only screening tools were used to identify cases and the prevalence among clinically confirmed cases was 14.8 (95% CI=14.6-15.0) *vs* 4.0 (95% CI=3.9-4.1 for those which used screening tools only. We calculated the pooled prevalence of studies that used the same screening tools. Only the TQQ had a sufficient number of studies to calculate the pooled prevalence which was 11.9 per 1000 population (95% CI=10.7-13.0).

**Figure 3.**
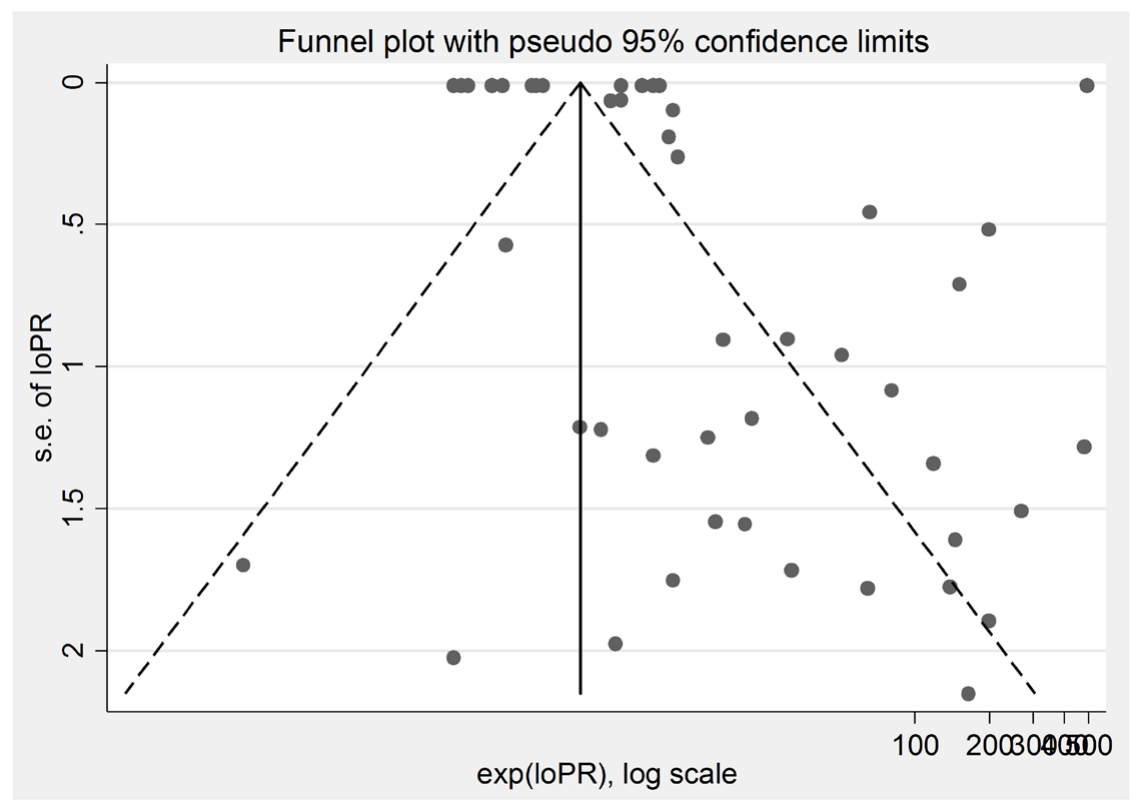
A funnel plot showing bias in published studies.

The random effect model for all studies was associated with a very high between-study heterogeneity (p = 0.000, I ^2^=99.9%). Some studies plotted outside the funnel outline in the meta-funnel analysis ( Figure 3) suggesting publication, reporting and selection bias.

### Factors explaining variation in documented overall prevalence

We assessed several factors in the univariable and multivariable models and six appeared to explain the highest variation in the documented median prevalence in terms of prevalence ratios. The type of NDD (whether a mental disorder or neurological disorder) was significantly associated with the greatest prevalence ratios in the multivariate analysis, (PR=2.6 (0.6-11.6, p<0.05). Table 4 summarizes these findings.

**Table 4.** Heterogeneity and factors contributing to heterogeneity.

Factor	Univariable analysis			Multivariable analysis		
	Prevalence ratio (95%CI)	P value	Heterogeneity (%)	Prevalence ratio (95%CI)	P value	Heterogeneity (%)
Region (as defined by the United Nations regional groups)	1.2 (0.6-2.3)	0.4	0.3	0.9 (0.5-1.9)	0.9	1.6
Condition (mental or neurological)	2.9 (0.7-12.3)	0.1	4.7	2.6 (0.6-11.6)	0.0	1.6
Setting (rural, urban or mixed)	0.8 (0.4-1.4)	0.5	2.8	0.6 (0.3-1.1)	0.2	1.6
Year	1.2 (0.7-2.3)	0.8	2.2	1.1(0.5-2.3)	0.9	1.6
Quality score (%)	1.0 (0.9-1.0)	0.1	3.2	1.0 (0.9-1.0)	0.3	1.6
Case identification method (clinically confirmed *vs* screening tool only)	1.6 (0.5-4.8)	0.4	0.2	2.0 (0.6-7.4)	0.4	1.6

### Prevalence per 1000 of individual domains of neurodevelopmental disorders

Most studies were on epilepsy, n=16 (35%), followed by hearing impairment, n=8 (17%), visual impairment, n=5 (11%) and ADHD, n=5 (11%). Behavioural/emotional problems had the highest prevalence of 362 per 1,000 (95% CI=337.0-387.0) (based on 2 studies), while one study on mental disorders reported a prevalence of 232 (95% CI=199.0-268.0) per 1,000. ADHD had a prevalence of 61 (95% CI=54-69), epilepsy 8 (95% CI=7.8-8.2) and ASD 0.6 (95% CI=0.5-0.6) per 1000 ( Table 5).

**Table 5.** Mean Prevalence/incidence of individual neurodevelopmental disorders.

Condition	Number of studies reporting the condition (N=46)	Total sample size N=2740728	Mean prevalence per 1000 (95% CI)	Mean Incidence per 100000 (95% CI)
ADHD	5 (10.9%)	3897 (0.1%)	60.8 (53.5-68.8)	-
Behavior problems	2 (4.3%)	1458 (0.1%)	362.1 (337.4-387.4)	-
Cerebral palsy	3 (6.6%)	777343 (28.4%)	1.6 (1.4-1.6)	-
Epilepsy	16 (34.8%)	652240 (23.8%)	8.0 (7.8-8.2)	447.7 (415.3-481.9)
Hearing impairment	8 (17.4%)	53756 (2.0%)	11.4 (10.5-12.4)	-
Motor impairments	1 (2.2%)	1022 (0.0%)	14.6 (8.2-24.1)	-
Neurological dysfunction	2 (4.3%)	5171 (0.2%)	75.2 (68.2-82.8)	-
Visual impairment	5 (10.9%)	62032 (2.3%)	177.8 (174.8-180.8)	-
Learning disabilities	1 (2.2%)	2036 (0.1%)	80.0 (68.6-92.7)	-
Neurodevelopmental delay	1 (2.2%)	2043 (0.1%)	32.8 (25.5-41.5)	-
Other mental disorders*	1 (2.2%)	611 (0.0%)	232.4 (199.5-268.0)	-

### Incidence of neurodevelopmental disorders

Three studies reported the incidence of epilepsy with a mean annual incidence of 447.7 (95% CI 415.3-481.9) per 100,000 ^31, 35, 56^. The study characteristics of all studies included in the meta-analysis are reported on Table 2.

### Regional distribution of neurodevelopmental disorders

The studies were distributed as follows: Africa n=16 (31.4%) (77.6%), Asia-Pacific n=19 (37.3%), Western-European n=7 (13.7%), Latin-America n=7 (13.7%), Multisite n=2 (3.9%).

Asia-Pacific had the highest number of domains studied (N=8, 73%) followed by Africa (N=6, 55%) then Latin America (N=3, 27%). Latin America had the highest pooled overall prevalence per 1,000 for all NDD of 33.4 (95% CI=28.9-38.0), whereas Africa had the least 4.4 (95% CI=4.2-4.6). Epilepsy was the most reported condition in Asia and Africa. ADHD and hearing impairments most reported in South America.

Analysis of the settings of the studies (rural or urban), findings were available for 27 (57.4%) studies of which 15 (56%) were conducted in an urban setting, 10 (37%) in rural and 2 (6%) in both settings. The overall pooled prevalence in rural areas was 6.1 (95%CI 5.7-6.4) and was 2.1 (95%CI 2.1-2.2) per 1,000 in urban areas. We provide a summary of regional findings of the prevalence of individual domains of neurodevelopmental disorders in Table 6.

**Table 6.** Regional summary of spectrum of neurodevelopmental disorders.

NDD	Asia-Pacific (N=2122324)	Africa (N=277897)	Latin America (N=10354)	Mixed (N=330153)
Pooled overall prevalence of all NDD (per 1000) and their corresponding 95% **CI**	7.5 (7.4-7.6)	4.4 (4.2-4.6)	33.4 (28.9-38.0)	9.4 (9.0-9.7)
Mean prevalence per 1000 for individual neurodevelopmental disorders and their corresponding 95% CI				
Autism spectrum disorders	0.6 (0.5-0.6)	-	-	-
ADHD	80.7 (67.1-96.1)	62.5 (35.4-101.0)	47.9 (39.4-57.6)	
Epilepsy	6.7 (6.1-7.3)	3.9 (3.7-4.2)	5.5(2.0-12.0)	9.4 (9.0-9.7)
Behavioural/emotional problems	362.1 (337.4-387.4)	-	-	-
Cerebral palsy	1.6 (1.5-1.6)	-	-	-
Learning disability	-	80 (68.6-92.7)		-
Hearing impairments	8.1 (7.3-8.9)	125.8 (105.0-149.1)	45.4 (29.9-65.8)	-
Visual impairments	333.6 (328.5-338.7)	-	-	-
Motor impairments	-	14.7 (8.2-24.1)		-
Other mild neurological impairments	75.2 (68.2-82.8)	-	32.8 (25.5-41.5)	-
Other psychopathologies	-	232.4 (199.4-268.0)		-

These results do not include studies from Turkey which is the only country in the Western European category because the studies were too few to provide a pooled estimate.

### Risk factors for neurodevelopmental disorders

Risk factors were reported in 13/51 (28%) studies included. Perinatal complications were the most prevalent risk factors across the NDDs. They were as significant in four out of the five (80%) conditions for which risk factor data was available. The highest median odds ratio (OR=9.4 (IQR 4.9-13.8) for perinatal complications was on participants with hearing impairments. History of febrile seizures was significantly associated with epilepsy OR=2 (95%CI 1.7-10.8), hearing impairments OR=5.6 (95%CI 4.7-9.0) and mild neurological dysfunction OR=6.7 (95%CI 2.1-25.5). Environmental factors such as parental smoking and a history of febrile illness were also prevalent risk factors. Table 7 summarizes other risk factors data available from eligible studies.

**Table 7.** Risk factors for neurodevelopmental disorders and the corresponding median odds ratios with interquartile ranges.

	No. of studies (total =13 studies)	Epilepsy	Hearing impairment	Mild neurological dysfunction	Cerebral palsy	Psychopathology
Congenital malformations and injuries of the head	3	2.0 *	9.4 (4.9-13.8)	-	-	-
Family history	6	2.8 (1.7-4.0)	5.1 (2.9-7.3)	-	-	-
Environmental factors such as parental smoking and families with substantial psychosocial stress	4	5.5 (1.8-8.6)	0.3 (0.2-5.1)	-	-	1.7 * (95% CI 2.76-7.52)
Seropositivity to cysticercosis	1	4.2 * (95% CI 1.6-11.2)	-	-	-	-
Sex male	2	1.9 (1.5-2.3)	-	-	-	-
Perinatal complications	8	2.8 (2.2-10.2)	-	1.1 * (95% CI 1.1-1.2)	6.5 *(95% CI 4.4-9.3)	-
History of a febrile illness	5	2.0 (1.7-10.8)	5.6 (4.7-9.0)	6.7 * (95% CI 2.1-25.5)	-	-
History of snoring	1	6.5 * (95%CI 4.5–9.5)		-	-	-
Non febrile illnesses such as jaundice	2	-	5.6 (0.2-15.8)	-	-	-
Maternal complications	1	-	0.2 (0.1-0.2)	-	-	4.6 * (95% CI 2.76-7.52)

*Only one study reported this finding hence we provided the confidence interval from this study The overall median prevalence per 1000 for neurological impairments was 13.0 (IQR= 6.1-45.0) and the mean was 47.5 (95% CI=6.5-101.6). The **pooled** median prevalence estimate for neurological impairments is 11.1(95% CI=10.7-11.5) **Snoring when caused by upper highway obstructing may be associated with poor oxygen perfusion in the brain. Subsequent brain damage may lower seizure threshold eventually leading to epilepsy.

## Discussion

This review provides an estimate of the burden of NDD and associated risk factors in LAMIC. Only 51 eligible studies reported the epidemiology of NDD, with a wide range of prevalence or incidence estimates for each condition. This indicates that in many LAMIC, there is a paucity of data on even the most basic epidemiology of NDD, particularly of mental health disorders. The wide range of prevalence estimates even within the same regions is comparable to that found in a review by Durkin ^61^. It may be due to methodological differences ^66^ perhaps because of the difficulties involved in diagnosing most NDD particularly mental disorders for which there were fewer studies. The age of the child can complicate detection of NDDs since some disorders only manifest later in life, and the tools for detecting other disorders are relatively insensitive during early life. Furthermore, since there is considerable co-morbidity between these conditions complicating the estimates of the burden. Few studies reported risk factors for NDD with perinatal complications being the commonest risk factor for all NDD and febrile seizures for neurological disorders such as epilepsy.

Most studies were from Asia-Pacific; Africa and Latin America were under-represented. Although this may have affected the overall prevalence estimate, Polanczyk *et al.* in their review on ADHD demonstrated that geographical locations do not greatly influence prevalence outcomes ^66^. While the pooled estimates were comparable between Asia, Africa and Latin America, there were very few studies from the latter two continents. The minimum-pooled prevalence for all NDD was 7.5 per 1000, being higher for neurological disorders (11.3/1000) than for mental disorder studies (3.2/1000). This may be because of overrepresentation of studies on epilepsy, which is more widely studied in LAMIC. The estimates for mental disorders observed in this review are unexpectedly low, perhaps because detection of mental disorders such as ADHD and ASD is poor in LAMIC due to lack of tools and expertise for Measuring neurodevelopment in low-resource settings ^67^ and also because of some children dying early before diagnosis ^68^. In addition, surveys conducted in very young children may not detect ADHD. Prevalence of NDD is higher in rural areas compared to urban areas; which is consistent with previous studies of epilepsy ^69^ suggesting that risk factors might be more common in the rural areas. There was substantial differences between studies heterogeneity in the pooled estimates. The prevalence showed substantial variation between individual NDDs, being highest for visual impairment and lowest for ASD. The high heterogeneity observed for visual impairment may be related to the variability from the number of eligible studies included compared to ASD, but also to lack of standardised assessment. Only three studies documented incidence estimates and we could therefore not pool the findings.

This review shows that the burden of NDD is not precise and is probably greater than we have estimated. For instance, a robust study from rural Kenya utilising a demographic surveillance system on neurological impairments and disability had a much higher estimate (67/1000) than the one presented in this review ^13^. The low estimates from the review demonstrate that studies of individual conditions may not provide the true burden of NDD. A comprehensive study design approach to studying all NDD is important since these conditions overlap, and may be reliably screened together with a group of questions collated in one tool ^70^. The comprehensive screening approach would have important public health implication since many NDD overlap and the associated sequelae may be addressed by similar interventions.

The study showed disproportionately many studies of neurological impairments which may have skewed the overall pooled estimates. While some neurological impairments overlap with NDD ^71– 73^ a substantial proportion of common NDDs such as ADHD and emotional problems present without neurological comorbidities. The multivariate meta-regression analysis showed that neurological studies might have influenced the estimates, compared to mental disorder studies. Visual impairments, which are easier to detect, were the commonest NDD, perhaps also contributing to the high prevalence of neurological impairments ^74^. The paucity of mental disorder studies in these poor regions of the world may be related to the challenges in identifying these conditions such as lack of child and adolescent psychiatrists ^75, 76^. In ADHD for instance, studies relied on reports from teachers and parents to make diagnosis ^51^. It is difficult to translate these reports into valid and reliable case definitions because of the varying definitions of “normal behaviour” in different societies. However with the current success in local adaptation of tools for assessing behavioural ^77^ and developmental disorders ^78^ quality studies on mental disorder conditions such as ADHD and ASD should be possible in poor regions of the world.

The low prevalence of mental disorders is likely contributed by ASD. The prevalence of ASD is much lower than the burden documented in literature, suggesting possible under recognition of ASD in LAMIC particularly Africa. A recent review of ASD in sub-Saharan Africa found only one study on the prevalence of ASD ^79^. On the contrary, other mental disorders may be easily recognised and assessed, for example, behavioural/emotional problems were reported in 36%, ADHD in 6% and other mental disorders in 23%, albeit all were based on less than five studies. It is likely that there are sporadic low-quality studies in LAMIC that are not published or are placed in unindexed journals, based on the evidence of publication bias from the funnel plots. More robust studies on mental disorders in children are needed in LAMIC. The identification of NDD in poor regions is becoming easier following the advent of cheap and easy assessment approaches including the mental health gap action program intervention guide ^80^. Tools such as WHO’s Ten Questions Screen can be used to screen those to be prioritised for diagnosis of NDD ^35, 81, 82^.

Few studies reported several risk factors ( Table 4). Perinatal complications ^21^ and family history of febrile seizures ^26, 35, 46, 83^ were common across a different number of NDD, particularly epilepsy. The role of perinatal complications in the risk of neurological conditions is recognised in previous studies ^83^ and improvement in obstetric services may be helpful. Family history of seizures was associated with neurological disorders in rural Kenya ^13^. Family history of seizures may represent genetic susceptibility or shared environmental factors for NDD, the later is supported by the high incidence of febrile infections in these regions. While environmental factors such as parental smoking are important in in mental health problems in children, few NDD studies from LAMIC investigated this factor. Gene-environment interactions should be explored as the risk for NDD in these poor regions of the world. Some of the risk factors mentioned have a higher incidence in LAMIC than in high-income countries, and could have an additive interaction effect with each other ^84– 86^ which probably explains the higher burden of NDD in the former parts of the world. Other risk factors such as fetal alcohol exposure which has been shown to have a high burden in some LAMIC ^87, 88^ and which result in neurodevelopmental impairments such as intellectual disability were not explored in the included studies and should be examined in future studies.

### Limitations

There were methodological differences and lack of use of standardized measures to assess NDD in most studies. To mitigate the effect of methodological differences on the prevalence estimates, we conducted a sub-analysis of prevalence estimates for studies that used the same methods of case ascertainment. Additionally, the pathophysiology of individual NDD varies widely and this limits the generalizability of intervention strategies. For example, whereas biomedical interventions such as medications and surgery may be more helpful in neurological impairments, alternative interventions such as behavioural therapy may be more helpful for mental health disorders. Subjective methods such as reports from teachers and parents were used to assess for the presence of impairments. This limits the reliability of the estimates provided in this study. The effect of sex on NDD could not be explored since prevalence results were not aggregated based on sex, despite evidence of male/female propensity in some NDDs such as ASD. We did not separate crude from adjusted estimates therefore the estimates we have provided may still be under estimates. Currently, there is no standard validated tool for assessing quality of evidence presented in observational studies hence, although we appraised the studies included in our meta-analysis, there may still be methodological limitations. Studies on neurological impairments such as epilepsy, which have lower prevalence than other mental disorders in other parts of the world, were overrepresented in the sample and that influenced the overall prevalence estimate. The estimates of ASD were lower than reports from high-income countries, which may have lowered the overall estimates of NDD. Lack of data on the severity of the NDDs limits the clinical implications of this study. Although NDD manifests early in development, delayed diagnosis in many LMIC may have delayed detection of these disorders at the time of the study. Some countries may have transitioned to high-income countries based on the World Bank classification of Economies and this may change the estimates provided in this study. For the studies where prevalence was not reported, we calculated it as a proportion cases over the total study sample. This method may have resulted in underestimation of the prevalence since there was no background information to adjust calculated prevalence for attrition and sensitivities of screening tools.

## Conclusions

This review indicates that the burden of NDD in LAMIC is considerable, but there is lack of reliable epidemiological data on some NDD such as ASD which may underestimate the true burden of NDD in LAMIC. Screening for all NDD in epidemiological surveys is recommended to provide reliable estimates for planning purposes e.g. to inform resource allocation towards the rehabilitation of affected children. Mental disorders such as ADHD and ASD were rarely reported, and more studies particularly in Africa and Latin America are required to provide reliable estimates since neurological conditions such as epilepsy usually have conserved estimates compared to mental disorders. The risk factors investigated were few with the role of perinatal complications and history of febrile seizures being consistent with previous studies. Studies considering all potential risk factors are required to inform preventive interventions aimed at mitigating the risk factors for neurodevelopmental disorders.

## Data availability

Final dataset for the systematic review is available on OSF: http://doi.org/10.17605/OSF.IO/9E2WY ^89^

Data are available under the terms of the Creative Commons Zero "No rights reserved" data waiver (CC0 1.0 Public domain dedication).

## Supplementary material

Supplementary File 1: PRISMA checklist.
