# Supplementary material for: Burden of neurodevelopmental disorders in low and middle-income countries: A systematic review and meta-analysis
Source: Wellcome Open Res. 2018 Mar 14;2:121. Originally published 2017 Dec 29. [Version 3] doi: 10.12688/wellcomeopenres.13540.3 (PMC5964629; doi:10.12688/wellcomeopenres.13540.3)
Supplement: Peer review history

# Peer review: Referee response for version 3

**DOI:** 10.21956/wellcomeopenres.15516.r33282

**Published:** 2018-06-08

**Authors:** Tina M Slusher, Jean-Baptiste Le Pichon

**Affiliations:** 1 City School of Medicine, University of Missouri-Kansas, Kansas City, MO, USA; 2 Department of Pediatrics, University of Minnesota, Minneapolis, MN, USA

In 2006 the World Health Organization published its first global report on neurological diseases. ^1^ In this publication they noted that, as a direct result of decreased mortality from infectious disease, there was a rising burden of neurological disorders worldwide. This new awareness for neurological diseases arose from the herculean work done and made public by the Global Burden of Disease Study in 2000. The importance of tackling neurological disorders was reemphasized by the follow-up Global Burden of Disease Study published in 2015. ^2^ While these studies did not differentiate between neurological and neurodevelopmental conditions, they emphasized the growing importance of these disorders worldwide. For example, the prevalence of epilepsy for all ages has increased by 39.2% between 1990 and 2015. ^2^ However, while these studies were informative in better understanding the burden of neurological disease as a whole, the global impact of neurodevelopmental disorders (NDD) could, at best, only be inferred from these studies (the conditions reported included Alzheimer’s disease, Parkinson’s disease, migraine headaches, multiple sclerosis, stroke and epilepsy). Yet, and somewhat paradoxically, as infant mortality rates continue to decrease worldwide (by close to 50% since 1990 ^3^) it is likely that the burden of NDD is growing. Indeed, a large proportion of childhood deaths occurs in the neonatal period (first 28 days of life). As the number of children who survive the neonatal period increases it is likely that NDD, as a result of challenges survived during this critical period, will increase in frequency.  It is therefore essential to gain a better understanding of the magnitude and distribution of children affected by NDD. We cannot hope to start addressing this problem until we better understand the scope it represents. The epidemiology of NDD in low and middle-income countries (LAMIC) is currently largely inferred from high income countries. Yet it is not clear how accurate these inferences are as the socioeconomic realties of LAMIC are very different from those of high income countries. As LAMIC are gradually emerging from a medical system geared primarily at acutely managing infectious illnesses, the burden of NDD is becoming more and more obvious. It is clear that medicine in these countries is undergoing a slow but fundamental shift toward managing the comorbidities of infants and children surviving medical conditions that would previously have been fatal. As this shift occurs, reevaluating the actual incidence and prevalence of NDD in LAMIC will be of critical importance in shaping therapeutic and interventional priorities. It is within this context that Bitta et al. undertake a metanalysis of available literature reviewing the incidence and prevalence of NDD in LAMIC ^4^.

 The authors start by defining NDD “neurodevelopmental disorders … typically manifest early in development and are characterized by developmental deficits that produce impairments of personal, social, academic, or occupational functioning” ^4^. These disorders include e both neurological (epilepsy, intellectual disability, hearing and visual impairments, and cerebral palsy) and neuropsychiatric disorders (autism spectrum disorders (ASD) and ADHD). This meta-analysis is conducted using the best possible tools for this sort of systematic review of the literature. It follows the Joanna Briggs Critical Appraisal tool ^5^ developed in 2014 by Munn et al. The authors were inclusive in a search that queried four large databases, including the African Index Medicus. They used a broad search strategy with a large number of inclusive terms to capture as much of the relevant literature as possible. The papers they reviewed were all conducted in LAMIC, had a population base less than 19 years old, (or were stratified by age, allowing for extraction of the less than 19 years old individuals) and only full research articles were included (reviews, case reports, etc. were excluded). 51 studies were found to meet the selection criteria. Of those 9 met the “high quality” criteria with the remainder meeting the “acceptable quality” criteria. The articles reviewed were published between 1987 and 2015. 16 studies were from the African continent, 5 from the Americas (mostly Brazil), 27 from Asia and 2 were from multiple sites. 10 of the 51 studies were designed to capture neuropsychiatric disorders, while the rest were geared toward neurological disorders (with 16 studies designed to study the epidemiology of epilepsy). The authors immediately note an important caveat, that many of the studies used the Ten Question Questionnaire (TQQ) tool. The TQQ has fairly good specificity for NDD, but according to one study ^6^, it has low sensitivity (70 to 80% except for epilepsy where the sensitivity was 100%) and very low positive predictive value (11 to 33%). Furthermore, this tool may not capture the actual number of patients with neuropsychiatric disorders such as ASD and ADHD.

 The results of this meta-analysis are noteworthy for several reasons. First and foremost is the very large variability between different regions of the world. The reported overall prevalence of NDD in Latin America 33.4/1000, but in Africa 4.4/1000 and in Asia 7.5/1000. This difference most likely points to the fundamental limitations to such studies in LAMIC where it is likely that NDD are underreported. Indeed, it is intriguing that the prevalence of NDD is highest in Latin America and lowest in Africa, yet Sub-Saharan Africa has the highest rates of poverty, neonatal mortality and malnutrition. ^3^ One explanation might be that Sub-Saharan Africa is still dealing with such high mortality rates that they are not yet experiencing the full onslaught of NDDs, as many of the children most at risk for NDDs do not survive infancy. Another major issue relates to the ability to report NDDs in low-income countries. This was nicely highlighted in a recent meta-analysis on the burden of severe neonatal jaundice ^7^. This study highlights the difficulty in obtaining such data in LAMICs.  Despite numerous hospital-based studies highlighting the magnitude of the problem of severe neonatal jaundice in LAMICs, there were only 4 LAMICs that had population-based results. It is also instructive to compare the rates of NDDs reported in this systematic analysis to the available data for developed countries. Prevalence rates for many NDDs are readily available from the Centers for Disease Control and Prevention (see table). It is rather surprising to note how similar the numbers from the USA are to those reported in this study, notably for ADHD, cerebral palsy, epilepsy, learning disability and neurodevelopmental delays. This is likely a result of many different factors, including the difficulty at gathering this information in LAMICs, but also the differences in medical care. As noted above, improved medical care paradoxically associates with increased numbers of patients with NDDs (for example premature infants less than 30 weeks’ gestation are unlikely to survive in LAMIC, but are at highest risk for NDDs in high-income countries where survival of these infants is becoming routine). It is also important to note the 10-fold higher rate of reported of behavioral problems and hearing impairments. Why behavioral problems might be higher in LAMICs is not readily apparent but worth exploring. As pointed out by Galler et al, while most children now survive, early malnutrition is associated with continued neurodevelopmental deficits including behavior problems. ^8^ Also worth noting is the higher prevalence of hearing impairments. One could postulate that infectious diseases such as CMV and rubella and the high incidence of severe neonatal hyperbilirubinemia in many LAMICs may contribute to the higher prevalence of hearing loss noted in this review. ^9^^-^^11^

 What becomes clear on reading this meta-analysis is that much more data is needed to start understanding the scope of the problem of NDDs in LAMIC. Furthermore, while greater numbers are needed, one might argue that information as to the etiology of these disorders will be as important, if not more, in forming an actionable impression. In summary, this study is comprehensive and well conducted. It is inclusive in its criteria while abiding by the best practices of systematic reviews. Yet, the results are confounding. They leave the reader perplexed and wanting to know more. And this is perhaps the most important point that this study makes and we whole heartedly agree with the author’s concluding statements “The burden of NDD in LAMIC is considerable. Epidemiological surveys on NDD should screen all types of NDD to provide reliable estimates.”

Table 1: Comparison of NDD prevalence in LAMIC and USA

 For an annotated version of the manuscript please click here.

We have read this submission. We believe that we have an appropriate level of expertise to confirm that it is of an acceptable scientific standard.

## References

[1] World Health Organisation: Neurological Disorders: Public Health Challenges. *WHO Press*.2006;

[2] GBD 2015 Neurological Disorders Collaborator Group: Global, regional, and national burden of neurological disorders during 1990-2015: a systematic analysis for the Global Burden of Disease Study 2015. *Lancet Neurol*.2017;16(11) : 10.1016/S1474-4422(17)30299-5 877-897 10.1016/S1474-4422(17)30299-5 28931491PMC5641502

[3] Millennium Development Goals report 2014. *Midwifery*.2014;30(10) :1043-4 25353044

[4] BittaMKariukiSAbubakarANewtonC: Burden of neurodevelopmental disorders in low and middle-income countries: A systematic review and meta-analysis. *Wellcome Open Research*.2018;2: 10.12688/wellcomeopenres.13540.3 10.12688/wellcomeopenres.13540.3 PMC596462929881784

[5] MunnZMoolaSRiitanoDLisyK: The development of a critical appraisal tool for use in systematic reviews addressing questions of prevalence. *Int J Health Policy Manag*.2014;3(3) : 10.15171/ijhpm.2014.71 123-8 10.15171/ijhpm.2014.71 25197676PMC4154549

[6] Mung'ala-OderaVMeehanRNjugunaPMturiNAlcockKCarterJANewtonCR: Validity and reliability of the 'Ten Questions' questionnaire for detecting moderate to severe neurological impairment in children aged 6-9 years in rural Kenya. *Neuroepidemiology*.23(1-2) : 10.1159/000073977 67-72 10.1159/000073977 14739570

[7] SlusherTMZamoraTGAppiahDStankeJUStrandMALeeBWRichardsonSBKeatingEMSiddappaAMOlusanyaBO: Burden of severe neonatal jaundice: a systematic review and meta-analysis. *BMJ Paediatr Open*.2017;1(1) : 10.1136/bmjpo-2017-000105 e000105 10.1136/bmjpo-2017-000105 29637134PMC5862199

[8] GallerJRKoetheJRYolkenRH: Neurodevelopment: The Impact of Nutrition and Inflammation During Adolescence in Low-Resource Settings. *Pediatrics*.2017;139(Suppl 1) : 10.1542/peds.2016-2828I S72-S84 10.1542/peds.2016-2828I 28562250PMC5374755

[9] OlusanyaBOOkoloAA: Adverse perinatal conditions in hearing-impaired children in a developing country. *Paediatr Perinat Epidemiol*.2006;20(5) : 10.1111/j.1365-3016.2006.00733.x 366-71 10.1111/j.1365-3016.2006.00733.x 16911014

[10] OliveiraLGoulartBChiariB: LANGUAGE DISORDERS ASSOCIATED WITH DEAFNESS. *Journal of Human Growth and Development*.2013;23(1) : 10.7322/jhgd.50389 10.7322/jhgd.50389

[11] RoghaMMokhtariE: Study of the knowledge of pediatricians and senior residents relating to the importance of hearing impairment and deafness screening among newborns. *Iran J Otorhinolaryngol*.2014;26(75) :57-64 24744993PMC3989869

# Peer review: Referee response for version 3

**DOI:** 10.21956/wellcomeopenres.15516.r33128

**Published:** 2018-05-21

**Authors:** Zulfiqar A Bhutta

**Affiliations:** 1 SickKids Centre for Global Child Health, Hospital for Sick Children, Toronto, ON, Canada

This is an interesting and potentially important systematic review of literature from Low and Middle Income Countries (LMICs) on the burden of neurodevelopmental disorders. As described in the methods, the authors specifically sought population representative studies to derive prevalence and incidence data.

 Although the authors seem to have thrown the net wide, the search strategy and the terms therein does appear to be limited and one wonders therefore as to the completeness and hence, representativeness of the review. I looked for studies from Pakistan and while the one recent study by my group in Sindh was included, two important cohort studies from Punjab were absent ^1^^-^^2^. One wonders if help from a qualified librarian was sought in developing the search (and associated MeSH terms)?

 The study sample sizes vary greatly from less than 200 to several thousand, and a legitimate question would pertain to the inclusion of such small sample sizes in the analysis; these couldn’t be representative population samples. Some comment on these small studies would be warranted.  

 The other source of data might be from the Cohorts group, as well as recent follow up studies by the Saving Brains consortium, and one wonders if an overture was made?

 Finally, it would have helped to see a comparison with the GBD estimates for LMICs to compare envelopes as well as major diagnoses.

I have read this submission. I believe that I have an appropriate level of expertise to confirm that it is of an acceptable scientific standard, however I have significant reservations, as outlined above.

## References

[1] YaqoobMBashirATareenKGustavsonKHNazirRJalilFvon DöbelnUFerngrenH: Severe mental retardation in 2 to 24-month-old children in Lahore, Pakistan: a prospective cohort study. *Acta Paediatr*.1995;84(3) :267-72 778024710.1111/j.1651-2227.1995.tb13627.x

[2] GustavsonKH: Prevalence and aetiology of congenital birth defects, infant mortality and mental retardation in Lahore, Pakistan: a prospective cohort study. *Acta Paediatr*.2005;94(6) : 10.1080/08035250410025636 769-74 10.1080/08035250410025636 16188785

# Peer review: Referee response for version 3

**DOI:** 10.21956/wellcomeopenres.15516.r31921

**Published:** 2018-03-15

**Authors:** Carl C. Bell

**Affiliations:** 1 Department of Psychiatry, University of Illinois at Chicago, Chicago, IL, USA

Much better as it takes into account probably the biggest contributor (fetal alcohol exposure) to Neurodevelopmental Disorders in childhood.

I have read this submission. I believe that I have an appropriate level of expertise to confirm that it is of an acceptable scientific standard.

# Peer review: Referee response for version 2

**DOI:** 10.21956/wellcomeopenres.15269.r31063

**Published:** 2018-02-28

**Authors:** Carl C. Bell

**Affiliations:** 1 Department of Psychiatry, University of Illinois at Chicago, Chicago, IL, USA

I guess my comments were too subtle to get a response and I did approve the article, however the consideration of fetal alcohol exposure in the continents studied still did not get any mention.  I suspect I should have been more diligent in my comments, but it appears I get another chance to comment.

 May *et al* ^1^ found "In this low SES, highly rural region, FAS occurs in 93–128 per 1000 children, PFAS in 58–86, and, ARND in 32–46 per 1000. Total FASD affect 182–259 per 1000 children or 18–26%."

 Adnams CM.  The determinants of Intellectual Disability and related mental illness in Africa presented at 3RD Annual Malawi Mental Health Research Research and Development Conference in 2013 (I realize this was not published and technically may not have been grist for the mill in the paper) noted the problem of Intellectual Disabilities, considered by DSM-5 to be a neurodevelopmental disorder, in the continent of Africa.

 A publication on Intellectual Disability is available by Maulik PK, *et al* ^2^ which examines the prevalence of ID in some of the continents the authors of the article under consideration are studying.  It is my sense that Intellectual Disability is often a neurodevelopmental disorder and a common etiologic factor of this disorder is fetal alcohol exposure.

 Lange S *et al* ^3^ lists six studies done in South Africa and gives prevalence of FASD using WHO regional and global mean prevalence of FASD.

 There is another study on FASD in Malawi which I cannot locate at the moment, but I guess, to reiterate, I continue to have a concern that the issue of Fetal Alcohol Spectrum Disorders were not considered in this study.

I have read this submission. I believe that I have an appropriate level of expertise to confirm that it is of an acceptable scientific standard, however I have significant reservations, as outlined above.

## References

[1] MayPAde VriesMMMaraisASKalbergWOAdnamsCMHaskenJMTabachnickBRobinsonLKManningMAJonesKLHoymeDSeedatSParryCDHoymeHE: The continuum of fetal alcohol spectrum disorders in four rural communities in South Africa: Prevalence and characteristics. *Drug Alcohol Depend*.2016;159: 10.1016/j.drugalcdep.2015.12.023 207-18 10.1016/j.drugalcdep.2015.12.023 26774945PMC4724497

[2] MaulikPKMascarenhasMNMathersCDDuaTSaxenaS: Prevalence of intellectual disability: a meta-analysis of population-based studies. *Res Dev Disabil*.32(2) : 10.1016/j.ridd.2010.12.018 419-36 10.1016/j.ridd.2010.12.018 21236634

[3] LangeSProbstCGmelGRehmJBurdLPopovaS: Global Prevalence of Fetal Alcohol Spectrum Disorder Among Children and Youth: A Systematic Review and Meta-analysis. *JAMA Pediatr*.2017;171(10) : 10.1001/jamapediatrics.2017.1919 948-956 10.1001/jamapediatrics.2017.1919 28828483PMC5710622

# Response

**Published:** 2018-03-08

**Authors:** Mary Bitta

**Affiliations:** KEMRI-Wellcome Trust Research Program, Kenya

Dear Dr Bell

 Thank you very much for pointing out the absence of  fetal alcohol exposure as a risk factor for neurodevelopmental disorders in our study.We acknowledge that fetal alcohol exposure is an important risk factor for some NDD such as intellectual disability. We have now included this in our discussion on risk factors and have included the references that you suggested.

 Regarding the study by Maulik PK, some of the individual studies in the review which met our inclusion criteria have been included in our analysis.

 Kind regards

 Mary

# Peer review: Referee response for version 1

**DOI:** 10.21956/wellcomeopenres.14705.r30191

**Published:** 2018-01-29

**Authors:** Carl C. Bell

**Affiliations:** 1 Department of Psychiatry, University of Illinois at Chicago, Chicago, IL, USA

I read the article and find it wanting but that is from no fault of the authors, rather it is a fault of the lack of good epidemiologic data that is out there about China, Africa, and South America.  The author's make good points about the need for better surveillance of the problem of neurodevelopmental disorders.  They should be looking at issues of autism, intellectual disability, ADHD, speech and language disorders, specific learning disorders, and motor disorders which are the usual problems, but it occurs to me that they should also be looking for fetal alcohol exposure as in some places it is a serious problem that leads to the usual 6 previously mentioned.  There is even evidence emerging that fetal alcohol exposure leads to epilepsy.  Unfortunately, the epidemiologic data in the three continents of interest was not very robust.

I have read this submission. I believe that I have an appropriate level of expertise to confirm that it is of an acceptable scientific standard.

# Peer review: Referee response for version 1

**DOI:** 10.21956/wellcomeopenres.14705.r29379

**Published:** 2018-01-29

**Authors:** Jane R W Fisher, Thach Tran

**Affiliations:** 1 Jean Hailes Research Unit, School of Public Health and Preventive Medicine, Monash University, Melbourne, Vic, Australia; 2 School of Public Health and Preventive Medicine, Monash University, Melbourne, Victoria, Australia

People with neurodevelopmental disorders (NDD) experience not only the health consequences of the condition, but also limits to social and economic participation, stigma and marginalisation. Addressing them through prevention strategies, screening programs for early detection, comprehensive interventions, equity of access to services and legislative and policy environments about rights and opportunities reduces the burden on individuals and families, but these are not distributed evenly throughout the world. Most evidence about population prevalence has been generated in high-income nations.

 This systematic review with meta-analyses sought to establish the burden of neurodevelopmental disorders (NDD) in low- and middle-income countries and to identify factors associated with these. This has important potential to assist understanding of whether conditions are predominantly attributable to biological factors, and so occur at similar prevalence in all nations, or reflect external factors that vary among countries including health systems, access to health services and essential medicines, public health infrastructure, and human and gender-based development indicators.

 The systematic review has been conducted and reported with considerable technical proficiency. There are however aspects of the conceptualisation, methods, analyses and interpretation which in our opinion warrant re-consideration:

  

1. It is not clear that the definition of NDD provided is widely used or accepted, it is drawn from a single study, and not a more authoritative source. There is little debate that conditions like cerebral palsy, autism spectrum disorders and epilepsy have neurological origins. However, to include ‘behaviour problems’ which are well known to reflect experiences, including of maltreatment, reduces conceptual clarity.
2. The inclusion criteria are quite well described, but need more precision to enable replication. The definition provided is that NDDs ‘manifest early in development’. It is of particular concern, given the aim, that no age criterion was used and so, while purporting to report burden among children and adolescents, it is not clear that studies were limited to or had to report disaggregated data for participants of this age to be included.
3. All systematic searches for evidence from LAMIC have to include the names of each country and cannot assume that studies have used the World Bank Classification of Economies in reporting their data. In our opinion it is essential that this is corrected
4. The studies included in the review are not listed as references (a serious oversight) and so we cannot assume in checking them that we have identified the same papers. However, to claim that they are all of ‘neurodevelopmental disorders’ appears inaccurate. As examples, the study of ‘hearing impairment’ by Czechowicz et al in Peru concluded that the most common cause among children was untreated infections. The study of ‘visual impairment’ (Zainal et al) was a national survey in Malaysia, included participants up to the age of 96 years, and concluded that untreated cataract among older adults was the major contributing factor. Antisocial behaviour, aggression and fearfulness among children in Gaza (Mousa Thabet et al) were attributed to living in a war zone.
5. The related central concern is that the overall prevalence is reported as though it relates to one disorder. Most studies were of a single condition, but others reported combined prevalence estimates for several NDDs (for example, Arrda et al; Couper et al). The pooled prevalence is therefore difficult to interpret.  The meta-regression with this outcome therefore makes little sense. In our opinion consideration should be given to removing the meta-analysis.
6. Given the age ranges reported in the few studies that we selected to read, we do not know how the authors reached the conclusion that *The median age of participants was 10.4 years, with an interquartile range (IQR) of 8.8-10.8 years and a full range 0.7–19.0 years. *This needs to be explained clearly.
7. The inclusion criterion is that there is a ‘population denominator’, so it is unclear how in studies in which prevalence is not reported, it is calculated on the basis of proportion of cases in the sample. Please explain what biases this might have introduced.
8. There is a lack of definitional clarity about places. The term ‘Asia’ is used without a definition and this needs to be much more specific (e.g. South Asia, South East Asia, or Central Asia). It is not at all clear why it is thought relevant to report the findings by ‘continent’ (sometimes described in the paper as regions) when there are established regional groupings of countries, including the ones used by United Nations agencies that are widely known and would assist with generalisation.
9. It is not meaningful under a sub-heading *Regional distribution of neurodevelopmental disorders*, to report the proportions of ‘populations’ in different regions. Saying that countries are ‘under-represented’ does not explain this construct. The differences reflect the available research and not absolute numbers in these settings. Clarity should be improved.
10. In the Discussion there is little engagement with whether these estimates suggest that there are disparities in prevalence of NDD between low- and middle-, and high-income nations, but this is of key importance to the translation of this evidence, including where efforts to ameliorate this burden should be focused. This should be added.
11. Calling ‘mental disorders’ neurodevelopmental is questionable, in particular as the implications for interventions are quite different. There should be discussion of this.
12. Further clarifications include:
  - *due sampling error*.... ? due to sampling error;
  - what are *high stress families*?
  - What is the significance of a *history of snoring*?

We have read this submission. We believe that we have an appropriate level of expertise to confirm that it is of an acceptable scientific standard, however we have significant reservations, as outlined above.

# Response

**Published:** 2018-02-12

**Authors:** Mary Bitta

**Affiliations:** KEMRI-Wellcome Trust Research Program, Kenya

**RESPONSE TO REVIEWERS**

 We thank the reviewers for their very helpful comments and we have provided a point by point response to each comment.

 1.       It is not clear that the definition of NDD provided is widely used or accepted, it is drawn from a single study, and not a more authoritative source. There is little debate that conditions like cerebral palsy, autism spectrum disorders and epilepsy have neurological origins. However, to include ‘behaviour problems’ which are well known to reflect experiences, including of maltreatment, reduces conceptual clarity.

**Reply: We have now revised the reference cited for the definition of NDD and replaced it with the Diagnostic Statistical Manual Fifth Edition (DSM V) which is the original source of the definition. DSM V describes NDD as “A group of conditions with onset in the developmental period. The disorders typically manifest early in development, often before the child enters grade school, and are characterized by developmental deficits that produce impairments of personal, social, academic, or occupational functioning.”** **https://dsm.psychiatryonline.org/doi/abs/10.1176/appi.books.9780890425596.dsm01**

 The inclusion criteria are quite well described, but need more precision to enable replication. The definition provided is that NDDs ‘manifest early in development’. It is of particular concern, given the aim, that no age criterion was used and so, while purporting to report burden among children and adolescents, it is not clear that studies were limited to or had to report disaggregated data for participants of this age to be included.

**Reply: In our inclusion criteria, we have now specified that “We only considered studies with a sample population of <19 years or if results were stratified by age, and a population denominator for sample <19 years was provided”. We have also noted in the limitation section of the discussion that “These NDDs may have started early, but because of delayed diagnosis in many LMIC, they may have been detected much later at the time of the study.**

 2.       All systematic searches for evidence from LAMIC have to include the names of each country and cannot assume that studies have used the World Bank Classification of Economies in reporting their data. In our opinion it is essential that this is corrected

**Reply: We recognize the limitations of World Bank Classification of Economies criteria and have noted in the limitations section that this may have left out countries that were previously LAMIC but had transitioned into HIC during the study period.**

 3.       The studies included in the review are not listed as references (a serious oversight) and so we cannot assume in checking them that we have identified the same papers. However, to claim that they are all of ‘neurodevelopmental disorders’ appears inaccurate. As examples, the study of ‘hearing impairment’ by Czechowicz et al in Peru concluded that the most common cause among children was untreated infections. The study of ‘visual impairment’ (Zainal et al) was a national survey in Malaysia, included participants up to the age of 96 years, and concluded that untreated cataract among older adults was the major contributing factor. Antisocial behaviour, aggression and fearfulness among children in Gaza (Mousa Thabet et al) were attributed to living in a war zone.

**Reply: We have now added the references to the all the included studies.**

 4.       The related central concern is that the overall prevalence is reported as though it relates to one disorder. Most studies were of a single condition, but others reported combined prevalence estimates for several NDDs (for example, Arrda et al; Couper et al). The pooled prevalence is therefore difficult to interpret.  The meta-regression with this outcome therefore makes little sense. In our opinion consideration should be given to removing the meta-analysis.

**Reply: We acknowledge the difficulty in interpreting the pooled overall prevalence. Rather than stating that the pooled prevalence is for all NDD, we have now re-stated that the overall prevalence is for any NDD, to mean the presence of at least one NDD.**

 5.       Given the age ranges reported in the few studies that we selected to read, we do not know how the authors reached the conclusion that *The median age of participants was 10.4 years, with an interquartile range (IQR) of 8.8-10.8 years and a full range 0.7–19.0 years. *This needs to be explained clearly.

**Reply: We have now only reported the range of the median age reported in individual studies (where this was available).**

 6.       The inclusion criterion is that there is a ‘population denominator’, so it is unclear how in studies in which prevalence is not reported, it is calculated on the basis of proportion of cases in the sample. Please explain what biases this might have introduced.

**Reply: We have now noted in the limitation section that “This method may have resulted in underestimation of the prevalence since there may be no background information to adjust calculated prevalence for attrition and sensitivities of screening tools”.**

 7.       There is a lack of definitional clarity about places. The term ‘Asia’ is used without a definition and this needs to be much more specific (e.g. South Asia, South East Asia, or Central Asia). It is not at all clear why it is thought relevant to report the findings by ‘continent’ (sometimes described in the paper as regions) when there are established regional groupings of countries, including the ones used by United Nations agencies that are widely known and would assist with generalization.

**Reply: We thank the reviewers for this observation. We have now revised the regional data to reflect the UN regional groupings.**

 8.       It is not meaningful under a sub-heading *Regional distribution of neurodevelopmental disorders*, to report the proportions of ‘populations’ in different regions. Saying that countries are ‘under-represented’ does not explain this construct. The differences reflect the available research and not absolute numbers in these settings. Clarity should be improved.

**Reply: We agree with the reviewer that the proportions reported only reflect the available research rather than the absolute numbers in these settings. We have now deleted this section to avoid confusion.**

 9.       Calling ‘mental disorders’ neurodevelopmental is questionable, in particular as the implications for interventions are quite different. There should be discussion of this.

**Reply: We thank the reviewers for this point. We have acknowledged how the widely varying pathophysiology of the individual disorders affects intervention strategies in our limitations section. In particular we note that “Non-treatment interventions may be more useful in neurodevelopmental disorders, while treatment is more helpful in mental health disorders”.**

 10.   Further clarifications include:

 o *due sampling error*.... ? due to sampling error;

***Reply*** **: This was a typographical error and has been corrected.**

 o    what are *high stress families*?

**In this context we were referring to families undergoing psychosocial stress that results from factors such as poverty (and all the negative consequences of deprivation) exposure to negative life events such as natural disasters etc. In the light of this, the term high stress families has now been revised to read “families with substantial psychosocial stress”.**

 o    What is the significance of a *history of snoring*?

**Reply: Snoring when caused by upper highway obstructing which may be associated with poor oxygen perfusion in the brain. Subsequent brain damage may lower seizure threshold eventually leading to epilepsy. This information is now provided in the revised manuscript.**
